# Opportunities for strengthening infant and young child feeding policies in South Asia: Insights from the SAIFRN policy analysis project

**DOI:** 10.1186/s12889-017-4336-2

**Published:** 2017-06-13

**Authors:** Anne Marie Thow, Sumit Karn, Madhu Dixit Devkota, Sabrina Rasheed, SK Roy, Yasmeen Suleman, Tabish Hazir, Archana Patel, Abhay Gaidhane, Seema Puri, Sanjeeva Godakandage, Upul Senarath, Michael J. Dibley

**Affiliations:** 1 Menzies Centre for Health Policy, School of Public Health, University of Sydney, Sydney, Australia; 2 Food and Agriculture Organization of the United Nations, Kathmandu, Nepal; 3 Department of Community Medicine and Public Health, Institute of Medicine, Kathmandu, Nepal; 4 Health Systems and Population Studies Division, International Centre for Diarrhoeal Disease Research Bangladesh (ICDDR,B), Dhaka, Bangladesh; 5 Bangladesh Breastfeeding Foundation, Dhaka, Bangladesh; 6 Health, Education and Literacy Program (HELP), Karachi, Pakistan; 7 Maternal, Neonatal and Child Health Research Network(MNCHRN), Islamabad, Pakistan; 8 Childrens Hospital, Pakistan Institute of Medical Sciences (PIMS), Islamabad, Pakistan; 9 Lata Medical Research Foundation, Nagpur, India; 10 Datta Meghe Institute of Medical Sciences, Nagpur, India; 11 Department of Nutrition, Institute of Home Economics, University of Delhi, New Delhi, India; 12 Family Health Bureau, Ministry of Health, Nutrition and Indigenous Medicine, Colombo, Sri Lanka; 13 South Asia Infant Feeding Research Network, Colombo, Sri Lanka; 14 Faculty of Medicine, University of Colombo, Colombo, Sri Lanka; 15 School of Public Health, University of Sydney, Sydney, Australia

**Keywords:** Infant and young child feeding, South Asia, Policy content analysis

## Abstract

**Background:**

South Asian countries experience some of the highest levels of child undernutrition in the world, strongly linked to poor infant and young child feeding (IYCF) practices. Strong and responsive policy support is essential for effective interventions to improve IYCF. This study aimed to identify opportunities for strengthening the policy environment in the region to better support appropriate infant and young child feeding.

**Methods:**

We mapped policies relevant to infant and young child feeding in India, Pakistan, Bangladesh, Sri Lanka and Nepal, based on a common matrix. The matrix described potentially relevant policies ranging from high-level strategic policy documents to implementation-level guidelines. We analyzed the data based on themes focused on caregiver interactions with IYCF interventions: provision of correct information to mothers, training of frontline workers, enabling mothers to engage with service providers and strategic support for IYCF.

**Results:**

Policy support for IYCF was present in relation to each of the themes assessed. In all countries, there was support for nutrition in National Development Plans, and all countries had some level of maternity protection and restrictions on marketing of breast milk substitutes. Sectoral and implementation-level policy documents contained provisions for system strengthening for IYCF and for training of frontline workers.

**Conclusions:**

The key opportunities for strengthening IYCF policy support were in relation to translating strategic directives into implementation level documents; improving multi-sectoral support and coordination; and increased clarity regarding roles and responsibilities of frontline workers interacting with mothers. These findings can support efforts to strengthen IYCF policy at the national and regional level.

## Background

South Asian countries experience a persistent, high burden of child malnutrition, with one of the key contributing factors being poor infant and young child feeding (IYCF) practices [1, 2].

Effective interventions for IYCF are well established – key components are promotion of breastfeeding, strategies to promote complementary feeding and general supportive strategies to improve family and community nutrition [3–5]. For the best outcomes at a national level, such interventions need to be supported by appropriate government policies [6–9]. The World Health Organization (WHO) and UNICEF have identified policy as key to creating an enabling environment to support appropriate infant feeding, as part of meeting the United Nations targets for nutrition [10].

Policy support entails political or strategic support for appropriate IYCF practices, as well as specific support at the sectoral level, and implementation-level support. IYCF is a multisectoral policy issue, that requires coordinated policy action across sectors. The WHO/UNICEF IYCF Strategy highlights the need for policies to provide community-based strategies, communication campaigns, restrict marketing of breastmilk substitutes, provide maternity leave, and invest in training and capacity-building in exclusive breastfeeding protection, promotion and support [10]. Also needed is engagement by regional governance structures, including roles such as coordination, funding, and guidelines for policy development [5]. In line with this, there has been significant investment in regional adaptation and national implementation of policies supporting best-practice IYCF interventions.

However, the effect of policy is dependent on appropriate implementation (see, for example, Lipsky’s work on the effect of implementers on the translation of policies into outcomes [11]). A recent review of IYCF programs in six countries, including in Bangladesh and Sri Lanka, found that policy implementation depended not only on supportive provisions, but also on committed partners across sectors, advocacy, training and planning [12]. Nevertheless, comprehensive and contextually appropriate policy content is associated with improved IYCF outcomes [13, 14].

In the South Asian region, policy support for IYCF varies widely. The World Breastfeeding Trends initiative (WBTi) monitors policies to support breastfeeding based on the Global Strategy for Infant and Young Child Feeding [15]. The recent WBTi review indicated that while many countries in South Asia had a range of globally recommended policy documents in place, gaps in coverage and implementation remained [16–20]. Challenges identified in the WBTi review by the countries included in this study included: poor linkages and coordination between the Ministry of Health and other departments/Ministries [17, 19, 20]; inadequate maternity leave provisions [16–20]; and a paucity of targeted policy documents guiding comprehensive action on IYCF [18–20].

The aim of this study was to improve understanding of the range and types of policy support for IYCF across countries in South Asia. Previous research demonstrated differences in IYCF indicators across the five countries in the South Asia Infant Feeding Research Network (SAIFRN) [21, 22]. The SAIFRN policy content analysis presented here was part of a larger study, underpinned by theories of policy agenda setting and reform [23, 24], that examined IYCF policy content, the role of influential actors in IYCF policy making, and implementation of policies for IYCF counselling in each SAIFRN country. While policy content is only one dimension of the policy making process – which broadly includes issues of agenda setting, power, stakeholder influence and politics – policy content analysis is useful for grounding recommendations for advocacy in the current policy landscape, and for understanding the relevant existing policies with respect to a multisectoral policy issue (10, 11). Detail on the specific provisions in policies, lends greater scope for constructive recommendations regarding how these could be strengthened.

Given the potential importance of policy in shaping outcomes, this study was designed to explore existing policy support and thus underpin recommendations for improving support for IYCF. Policy for IYCF involves multiple sectors and actors, and this analysis of policy content was paired with an analysis of policy stakeholders [25]. Our objective was to assess strengths and opportunities in IYCF policy content across the five countries. By analyzing the data across countries, we were also able to identify opportunities for policy learning between countries, and potential for technical support by regional actors.

## Methods

This study was conducted by local SAIFRN research teams in Sri Lanka, India, Nepal, Bangladesh and Pakistan, with technical support from the University of Sydney authors. We analyzed policy content supporting IYCF from both a political perspective – considering indicators of whole-of-government support for IYCF – and through a ‘caregiver’ lens. This approach focuses on how specific provisions in policy support mothers and other caregivers to receive best-practice IYCF interventions. For example, through provision of IYCF counselling, though time (e.g. maternity leave) to access care providers, and through availability of education. Our definition of policy thus included nutrition, health, or multi-sectoral policies that have IYCF-relevant components.

We used a systematic approach to map policies that support appropriate IYCF in Bangladesh, India, Pakistan, Nepal and Sri Lanka. Based on the work of Buse et al., we considered “health policy” as embracing ‘courses of action (and inaction) that affect the set of institutions, organizations, services and funding arrangements of the health system but focused on public (government) policy’ ([26], p6).

### Data collection

The SAIFRN research team (across all countries) created a common matrix for data collection across countries, using mind mapping to identify relevant policy document types and areas that could incorporate support for improved IYCF [27]. The different levels of policy considered, included: strategic whole-of-government documents (e.g. national development plans and other policy documents that describe over-arching priorities for government), sector specific documents (e.g. Health sector policies that include provisions relevant to IYCF), and implementation level guidelines relevant to breastfeeding and complementary feeding (e.g. training or clinical protocols that guide implementation). We included policies from within and outside of the health sector, that indicated government support, or influenced caregiver access to best-practice interventions. Relevant sectors were identified as: health, child development, labour, central planning, social welfare and agriculture. Key issues included: IYCF Counselling; support for early initiation of breastfeeding, appropriate breastfeeding and appropriate complementary feeding; and whether statements of policy intent are supported/translated into implementation level documents.

Our analysis focused on the national level, as the focus of population-level policy recommendations. However, in Pakistan and India we also included subnational level policies from two Provinces and two States, due to devolution of policy responsibilities related to IYCF (to different degrees) in these jurisdictions. In these countries, significant responsibility for health and child development policies is situated in the subnational levels of government.

Data were collected in 2013–2014. We sourced documents from government websites, government archives, and through direct requests to Ministry of Health officials and other related Ministries (e.g. Child Development) and other relevant stakeholders. For each policy document, information was entered into an excel spreadsheet, including: name of Policy; year of release; any relevant endorsement (e.g. by Cabinet); any mention of nutrition and specifically IYCF; references to other relevant policy documents. Detail on country-level data collection is presented in other papers in this Supplement [28–32].

### Data analysis

The first phase of analysis was conducted at country-level. Each team extracted relevant text of the IYCF policies into a structured excel spreadsheet for policy content analysis, drawing on qualitative policy research techniques [33–35]. This analysis was based on the over-arching research question: How is IYCF supported at the policy level in [Bangladesh, India, Nepal, Pakistan and Sri Lanka]? The extraction focused on the following key domains that were identified during the development of the data collection framework:

1) strategic/high level policy support for IYCF;

2) provision of standardised information to mothers and carers;

3) training of health workers in IYCF;

4) enabling mothers to engage with health care workers (e.g. maternity leave);

5) [cross-cutting theme] translation of high-level policy statements into implementation-relevant documents;

Each country team coded the data using the domains as a structured protocol. Within each group of policies (above), we adapted narrative synthesis and descriptive analysis approaches for policy content analysis [36, 37]. Narrative/thematic synthesis involves describing the findings of an analysis or review using an integrated critical perspective. We used this to describe the content related to IYCF, within the umbrella of nutrition more broadly, for each policy document identified. Detail on country level approaches to analysis is presented in other papers in this Supplement [28–32]. Throughout this process (2013–2015), the research teams met every 2 months via teleconference to discuss the methods, emerging findings, and analytical approach.

The regional analysis presented here was conducted by the lead author, and reviewed by each country research team leader for accuracy. For this regional overview paper, country-level findings were analysed to identify sub-themes within each of the pre-determined themes, regarding how support for IYCF is operationalized in the region. The sub-themes identified are described under each theme in the findings, with detail on the different approaches in each country. The level of policy support was then summarized as Robust, Emerging or an Opportunity to strengthen policy support (Table 1), based on the level of content in policy documents and level of translation into operational/implementation level documents.

## Results and discussion

### Strategic support for infant and young child feeding

The policy mapping revealed significant strategic/political support for IYCF at a regional level (Table 1). The policy landscape reflected a high degree of recognition that appropriate IYCF is essential to good child nutrition and health, and to development. There was also strategic support for multisectoral collaboration. It is notable that in all countries the focus at strategic level was on breastfeeding, with relatively little mention of the importance of complementary feeding.

**Table 1:** Summary of policy strengths and opportunities to strengthen policy support across the region

Themes and subthemes	Bangladesh	India	Nepal	Pakistan	Sri Lanka	Opportunities to strengthen policy support
R – Robust policy support; E – Emerging policy support; O – Opportunity to strengthen policy support^a^						
General support for infant and young child feeding						- Clear strategic mandates for cross-sectoral action and collaboration on IYCF - Role definition in areas of shared IYCF responsibilities - Improved policy support for monitoring and evaluation - Specific support for complementary feeding
- IYCF as development priority (e.g. in National Development Plans)	R	R	R	R	R	
- Provisions for multisectoral coordination, at whole-of-government level	R	E	R	E	R	
- Strategic policy support for monitoring and evaluation	O	O	O	O	R	
- High level (i.e. whole-of-government level) support for breastfeeding	R	R	R	E	R	
- High level (i.e. whole-of-government level) support for complementary feeding	E	O	E	E	E	
Provision of correct information to mothers/caregivers						- Consistent references to agreed messages regarding IYCF, especially complementary feeding, within policy documents
- Consistent and comprehensive IYCF messaging	R	R	R	R	R	
- Policy support for counselling	R	R	R	R	R	
- Restrictions on information provision by actors with vested interests	R	R	R	E	R	
Training of frontline workers^b^ in IYCF						- More integration of complementary feeding modules into training - Coordination and standard for training - Monitoring and evaluation of training
- Comprehensive cross-sectoral support for training	E	R	E	E	R	
- Detail on implementation of coordinated training	E	O	E	O	R	
Enabling mothers/caregivers to engage with best practice interventions						- Maternity leave for non-government workforce - Ongoing enhancement of rural and urban access to health systems that are equipped to deliver IYCF intervention
- Provisions for maternity leave	E	E	E	E	R	
- Other forms of support for working mothers (e.g. creches)	E	O	E	O	E	
- Community-based support for caregivers to engage with best-practice interventions	E	R	R	E	O	
Other						- Potential to continue to adapt global recommendations to address specific national and sub-national challenges (e.g. particular vulnerable groups), and to engage with relevant policies from other sectors
- Food security and dietary diversity	R	R	R	E	R	
- Policy support for equity	E	E	R	E	R	

Sources: [28–32]

^a^The level of policy support was then summarized as Robust, Emerging or an Opportunity to strengthen policy support, based on the level of content in policy documents and level of translation into operational/implementation level documents

^b^We have used the term ‘Frontline workers’ to refer to those engaging with mothers and caregivers

#### IYCF as development priority

Strategic support for IYCF was present in National Development Plans (or equivalent strategy documents) in all five countries [28–32]. Very specific support was present in the 5 Year Plans of the Governments of India, Nepal and Bangladesh, which included details regarding the benefits of IYCF for health and development and noted specific interventions; and in key Planning and Development documents at Federal and Province level in Pakistan. A particular strength of the policy approach in Pakistan was the strong mandate at the national level to implement a life-course approach to nutrition throughout the health system [31]. However, the devolution of health responsibilities to Provinces in 2011 created challenges with ensuring that this was uniformly translated into sectoral and implementation documents across each Province. This highlights the additional challenges arising from multiple layers of government. This issue was less of a concern in India, where states also hold significant governance responsibilities, as there was more centralization of responsibilities for nutrition and child development [28]. Commitment for multisectoral approaches to nutrition policy has led Nepal to have strong strategic support for IYCF in recent development plans (13th and 14th Plan documents) [32].

#### Provisions for multisectoral coordination, at whole-of-government level

All countries also had some level of high-level multisectoral collaboration on IYCF, which had been formalized for Nepal, Bangladesh and Sri Lanka. Sri Lanka had instituted a Nutrition Coordination Division under the Ministry of Health, with the mandate of coordinating action of relevant sectors on nutrition including IYCF, which is now also a priority area for prevention of non-communicable diseases, as well as child nutrition more broadly [29]. Nepal had a formal committee to support action on IYCF, with multisectoral representation, under the Multi-Sector Nutrition Plan [32]. This formal engagement of stakeholders from Women/Children, Agriculture, Education, Water and Sanitation and Social creates constructive lines of communication across sectors, and may promote collaboration for integration of IYCF in respective sectoral interventions. In Bangladesh, there is a 13 ministry coordination system that was established to coordinate the multi-sectoral activities around nutrition. Limited multisectoral collaboration on IYCF policy and programmatic issues was seen as a challenge in other countries. For example, in Pakistan, the nutrition boards and committees with a mandate to advise federal and provincial governments on nutrition policy were focused on IYCF and health, and did not include representation from other sectors [31].

In Bangladesh and India, Ministries of Health and Ministries responsible for Child Development were both responsible for implementing children’s health and IYCF policy, compared to the other countries, in which the Ministry of Health was the primary policy agency [28, 30]. This seemed to create both strengths and challenges. A key strength of this approach was the multiplication of intervention points for children and their caregivers – in particular, through health and community services, as well as social welfare or child development initiatives. However, this split responsibility could also result in certain populations being overlooked. For example, in Bangladesh, there was strong strategic support for IYCF across the Ministry of Health and Ministry of Women and Children’s Affairs, but specific strategies for implementation were unclear, particularly for mothers and children in urban areas, where primary health service provision was the responsibility of the Ministry of Local Government, Rural Development and Cooperatives and implemented by Local Government Divisions [30]. Similarly, in India, there was strong strategic support for IYCF within child development policies, but the detail at implementation level was limited. In contexts where responsibility for IYCF policy issues is shared, our analysis suggests that clear lines of communication and the creation of specific forums for strategic planning and definition of roles would improve consistency for IYCF support within services to caregivers and children [28].

#### Strategic policy support for monitoring and evaluation

Sri Lanka was the only country with high-level strategic policy support for monitoring and evaluation IYCF, as part of provisions for all nutrition programmes [29].

#### Support for breastfeeding and complementary feeding

In all countries, there was a distinct policy emphasis on breastfeeding, with relatively little emphasis on complementary feeding [28–32]. While all countries have strong strategic as well as implementation level policy support provision in place for promotion, protection and support for breastfeeding through community based strategies, consistent messages through all communication channels, enforcement of strict law for breast milk substitute and training service providers; the policy support for complementary feeding is somewhat unclear in terms of interventions, approach and coordination among sectors. The implementation level policy support lacks the comprehensive and clear guidance for promotion of complementary feeding among mothers and caregivers.

### Provision of correct information to mothers/caregivers

Across all countries there was strong and varied policy support for provision of correct information to mothers and other caregivers (Table 1). This included public messaging and counseling underpinned by clear and consistent reference information regarding appropriate IYCF, and also policies to prevent dissemination of incorrect or biased information.

#### Restrictions on information provision by actors with vested interests

All countries had enacted the International Code of Marketing of Breastmilk Substitutes into domestic legislation, which restricts the provision of information by actors with vested interests (in particular, infant formula manufacturers) [28–32]. This formed an important component of the standardisation of information provided to mothers – and the source of such information – particularly regarding breastfeeding.

#### Consistent and comprehensive IYCF messaging

Public messaging regarding appropriate IYCF practices was mandated by policy in all countries [28–32]. There were multiple avenues for provision of information to mothers, with responsibilities split between actors in different ways, and there were different approaches to the use of ‘reference’ documents to underpin the messages. For example, in Pakistan, the messages developed for IYCF were endorsed by National Infant Feeding Board, and the national health policies contained clearly defined and consistent definitions of appropriate IYCF practices to underpin public messaging [31]. In Bangladesh and Nepal, messaging was mandated in the IYCF National Communication Framework [12, 14], and consistent messages were also provided in Child Development policies [30]. In Nepal and Sri Lanka, mandates for awareness campaigns and consistent messaging were outlined within a range of health as well as non-health sector policies [29, 32].

There were three opportunities to strengthen policy support for provision of correct information to mothers identified in the content analysis. In Pakistan, there was explicit policy support for provision of clear messages regarding appropriate IYCF practices to illiterate and less educated mothers, in the nutrition policy guidance notes [31]. However, it was unclear how this would be implemented. In Sri Lanka, a policy gap identified was the potential for a more explicit link with the education sector, regarding the incorporation of IYCF messaging into health related curriculum in schools [29] – an approach already implemented in Bangladesh [30]. In Pakistan, the roles and approaches for public messaging were clear, but policy support would have been stronger with inclusion of details regarding how, when and through whom to disseminate information [31]. In Nepal, IYCF messaging was not integrated into the National Health Communication Policy [32].

#### Policy support for counselling

In light of growing evidence for the effectiveness of counseling in enabling appropriate IYCF practices – both globally and in South Asia [8, 38] – it was encouraging to see clear policy support for IYCF counseling in all countries, and also across a range of sectors, as a means to ensure provision of correct information to mothers [28–32]. In Bangladesh, Sri Lanka and Nepal, there was a policy focus on counseling at all points of contact between mothers and health services [29, 30, 32]. In Sri Lanka this was supported by specific Protocols and Guidelines, and in Nepal and Bangladesh by specific implementation documents (e.g. Operational Plan for Community Based Healthcare), to ensure consistency across clinics [29].

In India, there was strategic multisectoral policy commitment to IYCF counseling, supported by a mandate in the National Development Plan for IYCF counseling in both the health and child development sectors [28]. Roles for shared implementation responsibility in these sectors were outlined by sectoral policies in both Child Development and Health, with a recent expansion of availability of counselling as the ICDS added counselling to its previous remit for provision of information. There was also evidence from Maharashtra and Andhra Pradesh that counseling was integrated into State level initiatives, such as Nutrition Missions. Both health and child development-based initiatives drew on consistent information provided in guidelines from the health sector.

### Training of frontline workers in IYCF

In all countries, training of frontline workers in IYCF was supported by policy, generally focused on capacity building and ‘in-service’ training (Table 1).

#### Comprehensive cross-sectoral support for training

The policy support for training varied from high level and sectoral support for both in-service and pre-service training in India, Sri Lanka and Nepal, to support for training only for Lady Health Workers in Pakistan. In Bangladesh, the Ministry of Health was responsible for training the rural health care workers at all levels in IYCF, but although the urban frontline health workers under the Local Government Ministry are supposed to deliver IYCF counseling, it is not clear how they are trained [28–32].

One recurring positive theme was a strong focus on breastfeeding and on counseling. For example, in Sri Lanka and Nepal, training materials were adapted from the WHO/UNICEF breastfeeding training manual and had a significant counseling focus [29, 32]. In addition, in Sri Lanka, specific training on counselling is provided as part of continuous professional development of health staff, and Public Health Midwife training includes a significant component on complementary feeding [29]. In India, there was policy support for capacity building related to counseling for frontline workers in both the health and child development sectors [28]. The split responsibility for IYCF between health and child development in India seemed to result in a strong emphasis on both infant and young child feeding, particularly in the child development sector – further strengthened by the addition of counseling to usual training of ICDS service providers [28].In Bangladesh there is good policy support for training frontline health workers in the public health sector on infant and young child feeding but less emphasis is given on training the urban workers [30]. In addition in various policy documents complementary feeding received less attention than breast feeding.

#### Detail on implementation of coordinated training

In India, Nepal, Pakistan and Bangladesh, the content analysis identified a need for coordinated training (potentially with a common training module) for all frontline workers engaging with mothers and young children – including clinical, public health, agricultural, child welfare and other frontline workers from public, NGO and private sectors [28, 30–32].

Another opportunity to strengthen policy supporting frontline worker training in IYCF, was the inclusion of monitoring and evaluation provisions for training. In all countries, there were limited provisions for monitoring, and it was thus difficult to ascertain the quality and scope of training provided.

### Enabling mothers/caregivers to engage with best practice interventions

Key avenues for policy support that enables mothers to engage with best-practice interventions were provisions for maternity leave, recommendations for support for working mothers to breastfeed, and strengthening access to health care systems (Table 1).

#### Provisions for maternity leave

All countries had legal provisions for maternity leave, which provided from 2 to 6 months of leave, and paternity leave, from 3 to 11 days, mainly for government employees [28–32]. For example, the Bangladesh Labour Act specifies 4 months of paid maternity leave for workers generally. For those working in public sector however, 6 months of maternity leave is mandated [30]. In India 6 months maternity protection is pr﻿﻿escribed in the Maternity Benefit Act of Ministry of Labour & Employment, Central Civil Services Leave Rules, the Employees State Insurance Act, and the National Food Security Act [28]. Overall, there were significant gaps in coverage, particularly in the informal sector, and also recommendations in Sri Lanka for maternity and paternity leave provisions to be reviewed [29]. This content analysis indicates that a review of coverage and implementation of maternity leave across the public and private sectors could significantly strengthen policy support that enables mothers to access care. It would also need to consider the significant proportion of women employed in the informal sector, and their ability to engage with health care and other services delivering IYCF interventions.

#### Other forms of support for working mothers

For employed women, there were also workplace-based interventions supporting appropriate IYCF practices. In India, Nepal and Bangladesh there was policy support for workplace crèches, breastfeeding breaks and breastfeeding rooms but no detail on implementation [28, 30, 32]. For example, in Nepal the IYCF Strategy and Plan of Action recommended provision of breastfeeding breaks and workplace day care centres [32]. In Sri Lanka, there were provisions for breastfeeding rooms in workplaces [29].

#### Community-based support

In all countries there were also mechanisms for community based support for mothers [28–32]. For example, in Nepal, mothers are referred to community breastfeeding support groups, coupled with specific targeting of ‘hard to reach’ mothers [29, 32]. In Pakistan the National Action Plan for Children and the National Program for Family Planning and Primary Health Care provided for community based IYCF support through Lady Health Workers [31]. However, this was also an area in which improved translation of policy recommendations into clear guidelines for implementation would strengthen policy support. For example, in Pakistan and Bangladesh, there was clear policy support for community based interventions through frontline workers, but other mechanisms for community based support were not described in policy documents or guidelines [30, 31].

In India, specific policy support for encouraging institutional delivery was identified as a mechanism for mothers to access community based IYCF intervention, for example, through the Baby Friendly Hospital Initiative (for which there was policy support in all countries) [28]. There was also integration of IYCF into village-level community interventions, with counseling also delivered through mobile clinics to support access for women in remote communities.

This analysis highlighted a need to strengthen policy support to enable mothers to engage with complementary feeding interventions – particularly working mothers. Possible avenues are further integration of complementary feeding interventions into child development interventions, or workplace-based interventions. For example, the Government of India’s National Plan of Action for Children recommends promotion of IYCF practices through nutrition demonstrations, training and counseling in workplaces [28].

### Other

Additional themes that emerged in the content analysis related to food security and dietary diversity, strategies for engagement of related sectors, and issues of equity.

#### Food security and dietary diversity

In Nepal, Pakistan, Sri Lanka, Bangladesh and India, there was clear support for IYCF arising from policy support for food security, particularly as a means for improving dietary diversity [28, 29, 31, 32]. For example, in Sri Lanka food security is supported in the National Nutrition Policy, and in India through the 2013 National Food Security Act, and in Bangladesh through Food Ministry policies [28, 29]. These policies were generally situated within the agricultural sector, and also indicated an opportunity to strengthen policy support for IYCF by strengthening links between health and agriculture policy with respect to complementary feeding. In Nepal, IYCF intervention has been integrated into other sectoral programmes, such as the child cash grant, and WASH interventions (Water, Sanitation and Hygiene) [32].

#### Policy support for equity

In all countries there was evidence of consideration of equity and coverage considerations with respect to IYCF. In Nepal and Sri Lanka in particular, many policies were framed around equity and social mobilisation, which led to specific targeting of vulnerable and ‘hard to reach’ families [29, 32]. In India, there was policy support for promotion of appropriate IYCF among Tribal peoples in Maharashtra state, a vulnerable group whose needs might differ from the general population [28]. In Pakistan, the needs of illiterate women were also identified within policy [31]. In Bangladesh women in special circumstances such as natural disasters and those suffering from HIV are mentioned specifically for intervention. This finding of the content analysis indicates successful adaptation of global recommendations to local situations. It also represents an opportunity to continue to strengthen policy support for IYCF by considering whether recent data has highlighted additional sub-populations vulnerable to poor IYCF practices that should be specifically targeted by policy.

## Conclusion

Overall, policy advocacy and global policy guidance over the past three decades appears to have resulted in a robust IYCF policy landscape, particularly with respect to breastfeeding, across South Asia. This policy content analysis focused on mothers/caregivers, and identified a wide range of policy support in existence in Bangladesh, India, Nepal, Pakistan and Sri Lanka. IYCF is a strategic development policy priority in all countries, with evidence for multi-sectoral engagement and cooperation between sectors in implementation. Specific strengths with respect to sectoral policies included widespread adoption of counseling as a best-practice intervention, and comprehensive and consistent messaging regarding appropriate IYCF practices.

This analysis identified several ways in which policy in South Asian countries could better support mothers to engage in appropriate IYCF practices. A cross cutting theme was the need for more consistent translation of sectoral policies and strategies into documents with details regarding implementation, including defining roles and responsibilities of different frontline workers – particularly those working in sectors outside of health. There was also little information available publicly regarding resourcing for IYCF in the policy documents we obtained.

The policy focus on breastfeeding indicated a need for more comprehensive policy support for complementary feeding and associated interventions. Given poor dietary diversity practices across the regions, the recent policy drive for linking nutrition with food system could have a crucial implication for the improved outcome for complementary feeding practices [39].

There were also opportunities identified to improve consistency and coverage of policies for maternity (and paternity) leave and training of frontline workers. In both cases, these policy areas were fragmented; in the case of maternity leave, significant sub-populations were excluded from policy provisions. With respect to training of frontline workers, in many cases there was incomplete coverage of either frontline worker types or training content areas, and only in Sri Lanka was there policy support for monitoring and evaluation of training, which likely contributed to the strong policy support regarding training in Sri Lanka.

Considering the analysis across the region as a whole also indicates some potential opportunities for further action by regional and global actors such as the WHO and UNICEF to support IYCF policy in South Asia. This would build on their significant contribution to IYCF policy making over the past four decades at the global (e.g. [5, 40, 41]) and regional level (e.g. [42]). Two key areas that would benefit from regional technical support are strategies to support integration of complementary feeding into policies, and also for enhancing multisectoral action. In addition, the recurring challenge of provision of maternity leave outside of government agencies suggests an opportunity for provision of guidelines and strategies regarding policy development and implementation at the supranational level. This would also benefit from continued input by the International Labor Organization, which holds responsibility for the International Maternity Protection Convention [43].

This research has provided an overview of the policy environment supporting IYCF in five countries in South Asia, with a particular focus on how mothers are supported to engage in best-practice IYCF. The strengths of the research include the systematic policy survey across all countries by local research teams with expertise in IYCF, and the inclusion of a wide range of policy documents, from high-level strategic documents such as National Development Plans, to sectoral documents and implementation-level guidelines and protocols. In the two countries with significant sub-national governance (India and Pakistan), we also included a sample of States/Provinces in our policy survey.

The limitations of the research include the single point of time for data collection, which did not allow the identification of changes in the policy environment over time. In addition, using a policy content analysis method did not allow us to assess the context in which a particular policy was drafted or implemented; as noted in the introduction, there are likely to be further, contextual, challenges to implementation. However, the findings of the SAIFRN stakeholder analysis presented in this Supplement do provide some further information on the wider context [25]. This study was also conducted by different research groups in each country, and although we endeavoured to minimize differences through regular teleconferences and meetings, there may have been some variation in the search strategy and analysis between study sites. We also were not able to include all States or Provinces in our policy surveys in India and Pakistan. With increasing decentralization, more information on sub-national variation in policy would be useful in identifying opportunities to strengthen policy in all countries. Although the researchers endeavoured to check that all policy documents relevant to IYCF in each country were included in the review, it is still possible that some documents were not identified as relevant. Further research on IYCF policy could address some of these limitations through: 1) a wider scope of research that includes implementation characteristics, 2) sub-national policy across all countries, and 3) additional data collection on government budget expenditure through budgetary analysis. There is also an opportunity for future research to analyse the context in which policy is formulated and implemented, and to compare policy support with the current outcomes for IYCF across countries.

Due to the common challenge of persistent child undernutrition across low and middle income countries, this research is likely to provide insights in other regions. First, the analysis has highlighted significant recognition of IYCF as a contributor to national development – but also that policy support for IYCF is not always accompanied by sufficient detail regarding implementation, which is likely to hamper outcomes. Second, monitoring and evaluation can easily be overlooked but is a critical component of strong policy support for IYCF. Finally, policy content analysis can be a helpful approach to underpin informed advocacy for stronger IYCF policy.

## Abbreviations

IYCF: Infant and young child feeding
SAIFRN: South Asia Infant Feeding Research Network
UNICEF: United Nations Children’s Fund
WHO: World Health Organization
